# Reduced Susceptibility of DNA Methyltransferase 1 Hypomorphic (Dnmt1^N/+^) Mice to Hepatic Steatosis upon Feeding Liquid Alcohol Diet

**DOI:** 10.1371/journal.pone.0041949

**Published:** 2012-08-08

**Authors:** Huban Kutay, Corie Klepper, Bo Wang, Shu-hao Hsu, Jharna Datta, Lianbo Yu, Xiaoli Zhang, Sarmila Majumder, Tasneem Motiwala, Nuzhat Khan, Martha Belury, Craig McClain, Samson Jacob, Kalpana Ghoshal

**Affiliations:** 1 Department of Molecular and Cellular Biochemistry, College of Medicine, The Ohio State University, Columbus, Ohio, United States of America; 2 Comprehensive Cancer Center, College of Medicine, The Ohio State University, Columbus, Ohio, United States of America; 3 Molecular, Cellular and Developmental Biology Program, College of Medicine, The Ohio State University, Columbus, Ohio, United States of America; 4 Center for Biostatistics, College of Medicine, The Ohio State University, Columbus, Ohio, United States of America; 5 Department of Nutrition, College of Medicine, The Ohio State University, Columbus, Ohio, United States of America; 6 Department of Medicine, University of Louisville and the Robley Rex Louisville VAMC, Louisville, Kentucky, United States of America; 7 Experimental Therapeutics Program, College of Medicine, The Ohio State University, Columbus, Ohio, United States of America; 8 Department of Pathology, College of Medicine, The Ohio State University, Columbus, Ohio, United States of America; Children’s Hospital Boston & Harvard Medical School, United States of America

## Abstract

**Background:**

Methylation at C-5 (5-mdC) of CpG base pairs, the most abundant epigenetic modification of DNA, is catalyzed by 3 essential DNA methyltransferases (Dnmt1, Dnmt3a and Dnmt3b). Aberrations in DNA methylation and Dnmts are linked to different diseases including cancer. However, their role in alcoholic liver disease (ALD) has not been elucidated.

**Methodology/Principal Findings:**

Dnmt1 wild type (*Dnmt1* ^+/+^) and hypomorphic (*Dnmt1* ^N/+^) male mice that express reduced level of Dnmt1 were fed Lieber-DeCarli liquid diet containing ethanol for 6 weeks. Control mice were pair-fed calorie-matched alcohol-free liquid diet, and Dnmtase activity, 5-mdC content, gene expression profile and liver histopathology were evaluated. Ethanol feeding caused pronounced decrease in hepatic Dnmtase activity in *Dnmt1* ^+/+^ mice due to decrease in Dnmt1 and Dnmt3b protein levels and upregulation of miR-148 and miR-152 that target both Dnmt1 and Dnmt3b. Microarray and qPCR analysis showed that the genes involved in lipid, xenobiotic and glutathione metabolism, mitochondrial function and cell proliferation were dysregulated in the wild type mice fed alcohol. Surprisingly, *Dnmt1* ^N/+^ mice were less susceptible to alcoholic steatosis compared to *Dnmt1* ^+/+^ mice. Expression of several key genes involved in alcohol (*Aldh3b1*), lipid (*Ppara, Lepr, Vldlr, Agpat9*) and xenobiotic (*Cyp39a1*) metabolism, and oxidative stress (*Mt-1, Fmo3*) were significantly (P<0.05) altered in *Dnmt1* ^N/+^ mice relative to the wild type mice fed alcohol diet. However, CpG islands encompassing the promoter regions of *Agpat9, Lepr, Mt1 and Ppara* were methylation-free in both genotypes irrespective of the diet, suggesting that promoter methylation does not regulate their expression. Similarly, 5-mdC content of the liver genome, as measured by LC-MS/MS analysis, was not affected by alcohol diet in the wild type or hypomorphic mice.

**Conclusions/Significance:**

Although feeding alcohol diet reduced Dnmtase activity, the loss of one copy of *Dnmt1* protected mice from alcoholic hepatosteatosis by dysregulating genes involved in lipid metabolism and oxidative stress.

## Introduction

Alcohol abuse is a leading cause of morbidity and mortality throughout the world. It is estimated that in the United States as many as 10% of men and 3% of women may suffer from persistent health problems related to the excessive consumption of alcohol [1]. Alcohol affects many organs of the body, notably the central nervous system and the liver. Excessive alcohol use may lead to acute and chronic liver disease, such as steatosis, acute and chronic hepatitis, cirrhosis and hepatocellular carcinoma (HCC). Epidemiologic studies have shown that heavy alcohol consumption promotes HCC in patients with viral hepatitis and in diabetics [2]. It is well established that ethanol causes oxidative stress, depletes glutathione, alters methionine metabolism and induces pro-inflammatory cytokines in the liver [3], [4]. Both genetic and epigenetic factors are thought to be involved in the predisposition of certain individuals to alcoholic liver disease (ALD) [5], [6].

**Table 1:** Sequences of RT-PCR primers.

Gene	Forward	Reverse
Agpat9	TGGAGGATGAAGTGACCCAGAG	AGAGGTAGCAGGAAGCAATAGCG
Lepr	CCCAGAGCCAAACTCAACTACG	TCCAGGAAGCACACTGTCTACCAG
Hsd3b5	GCAAAAGGATGGCTGAGAAGG	GTTGGCGATGGAGAATGTGG
Gpr110	TGTCACCCTTTGTCCCCTCTTC	TGGCTTCTCTTGGTCTGCTTCC
Pltp	CTTCCTGAGGGTATCAACTTCGTG	CGGTTCTTGTCAATCACTTCTCG
Vldlr	CGTTCTACTCAGTGTATCCCCGTG	ACAAAGTTTCTGGAGACGCAGC
Cyp2b10	TCCTGGTGCCCACAGACAAATC	TGCCGACAAAGAAGAGAGAGAGC
Cyp4a14	CACCAGATTCTTCTCACCATAGCC	ATTCAAAGCGGAGCAGGGTCAG
Cyp39a1	ATCCAAAAGATGGCTCCTGGCG	TGTTTCCGTCTCCACCACTTCC
Nr1i3	TCGGCTCCAAAGTCGGTTTC	CGTCATAGCAGACAGTTCCTCCAAG
Aldh3b1	TGCTATGTGGATGACAACTGCG	CGTGTGATGGCATTCTGTAGGG
Fmo2	CGGAAAACTAAAGGAGACACCAGC	ACTGCTCGGAGGGCTAAAGAAG
Nqo1	TGGCATCCTGCGTTTCTGTG	TTGGAGCAAAATAGAGTGGGGTC
Cbr3	GCTGGATGAAAAGAGGAAAGCG	CAGGAGAGCCAAGTAAACGGG
Fmo3	CCTATCCCGATGACTTTCCCAAC	CGTGCTTTTCAGTGGTGACTTCC
Nt5e	TCCTGTGCGACTCCAAAAGC	TGACTTGAATGAGAGCCGTGTTG
Cyp2c39	TCACTGGATGTTACAAACCCTCG	GGTCAATCTCTTCCTGGACTTTAGC
Mt1	CAACTGCTCCTGCTCCACCG	AAGACGCTGGGTTGGTCCG
PPARa	GTGAACACGACCTGAAAGATTCG	ATGTATGACAAAAGGCGGGTTG
Dnmt1	GCCTCGGTTCTTCCTTCTGG	CAGCCTGGAGCACACCAAAG
Dnmt3a	CAGCGTCACACAGAAGCATATCC	GGTCCTCACTTTGCTGAACTTG
Dnmt3b	TTGGTGATTGGTGGAAGCC	ACGGTTGTCGCCCTCCTTGG
Gapdh	TCCTGCACCACCAACTGCTTAG	TGCTTCACCACCTTCTTGATGTC
18S rRNA	TGACGGAAGGGCACCACCAG	TCG CTCCACCAACTAAGAACGGC

5-methyldeoxycytosine (5-mdC) is the most prevalent epigenetic mark that is essential for mammalian development [7], [8]. Methylation predominantly occurs at symmetrical CpGs in somatic cells although non-CpG methylation has also been detected in embryonic stem cells (ESCs). The major biological function of methylation is to inactivate the X chromosome in females, regulate genomic imprinting and maintain genomic stability [9]. Aberrations in DNA methylation are linked to different diseases including cancer [8].

**Table 2:** Sequences of primers used for COBRA of specific genes.

mVldlr - BSF	GGGGTTTTTTTTGTTTATTTTTTTT
mVLDLR - BSR	ACATCCCCTAACCCTTATCAATAA
mLEPR - BSF	GTAGTTATTTGAGTGGTTAGTGTTT
mLEPR - BSR	AAAAATATCCCACCTATATCCC
mAgpat9-BSF	TTTAAGGGGAGGAAGTAAGGAGAT
mAgpat9-BSR	TAAATATCAAAATCCCAAACAATCC
mCyp39a1-BSF	GTGTTATATGAGTTTTATTTGTATTAGGTA
mCyp39a1-BSR	AAAAATCCAAAAAACAACAACCA
mMyc-BS2531F	TTTGTTTTTTTTGTTGTGTTTTTTTTA
mMyc-BS 2729R	TTTCTTCCAAATATCCTCACTAAAC
mMyc-BS2891F	ATTTGGAGGAGATATGGTGAATTAG
mMyc-BS3159R	AAACCACTAAAAAATCAATACACTC
mMt1-BSF	GGTTTTTAGAATATTAAGTTGGGAT
mMt1-BSR	AACTAATAAAAACTTTTACAAAAAC
mPPARa-BS166F	TTTTGGGATTTTAAAGATTAGATTT
mPPARa-BS417R	ATAAAAAAACTACCCAAAATCACCC
mPPARa-BS951F	TGGAGATTTATAGTTATTGGAGAGG
mPPARa-BS1155R	AAAAACAACCAAAAAACCCAAC
mPPARa-BS814F	TAGTTTTAGGTGTTTAGGGGTTGG
mPPARa-BS976R	CCCTCTCCAATAACTATAAATCTCC

DNA methylation is established and maintained by three functionally related DNA methyltransferase (Dnmt) enzymes, namely Dnmt1, Dnmt3a, and Dnmt3b that are essential in mammals [10]. Methylated DNA is then recognized by methyl CpG binding proteins along with associated co-repressors that leads to silencing of the associated promoter [11], [12]. Unlike bacterial Dnmts, mammalian enzymes exhibit transcriptional repressor activity as well independent of their C-terminal catalytic activity by virtue of their relatively large N-terminal domain [13], [14].

**Figure 1:**
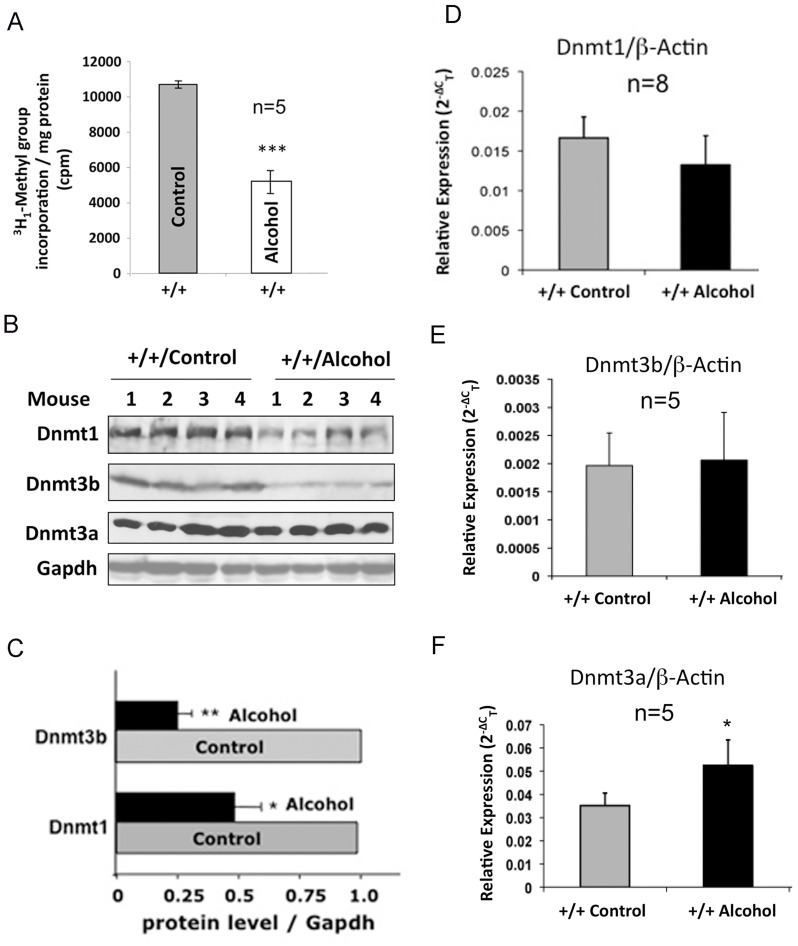
Hepatic Dnmtase activity is reduced in the wild type mice fed the Lieber-DeCarli alcohol diet compared to those fed the control diet for 6 weeks. **A.** Dnmtase activity was measured in triplicate in the liver nuclear extracts with poly(dI-dC) as the substrate and ^3^H_1_-Ado-Met as cofactor. The ^3^H_1_-methyl group incorporation in poly(dI-dC) was measured in a scintillation counter. Each sample was analyzed in triplicate. **B, C.** Hepatic Dnmt1 and Dnmt3b protein levels are reduced without significant changes in Dnm3a level in mice fed alcohol diet. Liver lysates (250 µg protein) were subjected to Western blot analysis with specific antibodies, signals were quantified using NIH ImageJ software and Dnmt levels were normalized to that of Gapdh. Dnmt1&3b levels in control livers were assigned a value of 1. **D–F**. qRT-PCR analysis showed significant increase only in Dnmt3a expression upon feeding alcohol diet. Hepatic cDNAs were subjected to qRT-PCR with gene specific primers using SYBR Green chemistry. Each sample was assayed in triplicates. Single, double and triple asterisks represent P-values ≤0.05, ≤0.01 and ≤0.005, respectively. Wild type mice are denoted by +/+.

The *in vivo* role of Dnmt1 in carcingenesis has been widely studied using hypomorphic mice instead of *Dnmt1^−/−^* mice that die during embyrogenesis [10]. *Dnmt1* ^N^ allele that express ∼50% of the wild type allele, protects tumor prone *Apc* ^Min/+^ or *Mlh1* ^−/−^ mice from intestinal neoplasia whereas it promotes leukemia in *Mlh1* ^−/−^ mice [15], [16]. The same hypomorphic allele appears to protect mice from prostate hyperplasia at early stages but promotes carcinoma at later stages [17]. In contrast, another hypomprphic (Dnmt1^chip/chip^) mice that express only 10% activity of the *Dnmt1^+/+^* mice develop spontaneous hepatocellular carcinoma with age [18], [19]. These observations clearly indicate that pathological manifestations in Dnmt1 hypomrphic mice is temporal and depend upon the nature of the hypomorphic allele and tissue or cell type.

**Figure 2:**
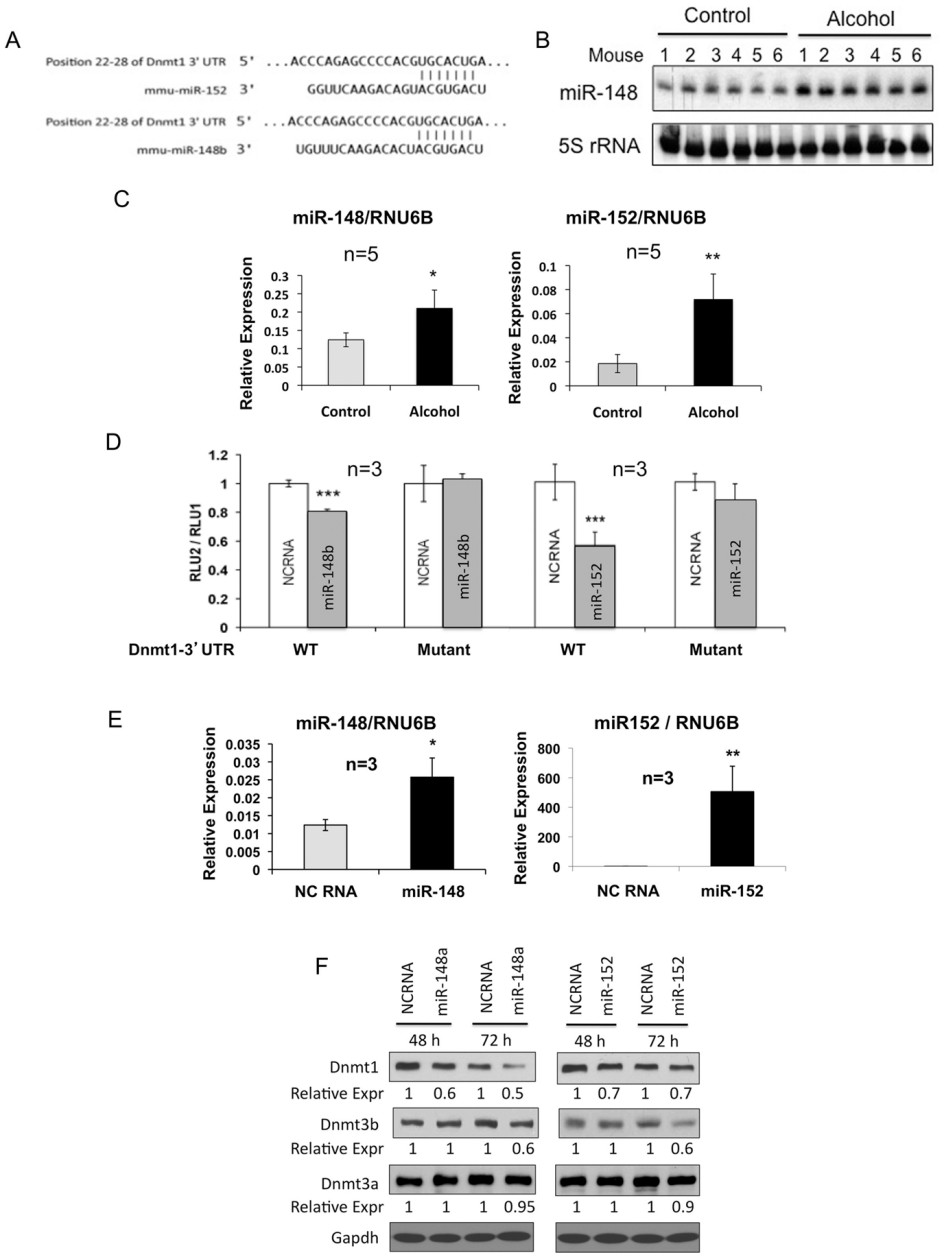
miR-148 and miR-152, which target Dnmt1 and Dnmt3b, are upregulated in the livers of the wild type mice fed the liquid alcohol diet. **A.** miR-148/152 cognate site predicted by TargetScan in the 3′-UTR of Dnmt1. **B.** Northern blot analysis demonstrated increased expression of miR-148 in mice fed alcohol. Total RNA (5 µg) was separated in 15% acrylamide-8 M urea gel, transferred to nylon membrane and subjected to Northern blotting using ^32^P-labeted anti-miR-148 oligo as probe, washed and subjected to autoradiography. The blot was rehybridized to 5S rRNA probe after stripping to demonstrate comparable RNA in each lane. **C.** qRT-PCR analysis confirmed upregulation of hepatic miR-148&152 in mice fed alcohol. miR-148/152 and RNU6B were measured in the liver cDNAs using Taqman probes and primers specific for each miRs and the data was normalized to RNU6B level. **D.** Dnmt1 is a validated target of miR-148&152. Hepa cells were transfected with psiCHECK2 vector harboring wild type or mutant Dnmt1 3′-UTR downstream of renilla luciferase coding region along with 50 nM miR-148b/152 mimic or scrambled RNA (NC RNA). After 48 h, renilla (RLU2) and firefly luciferase (RLU1) (expressed from the same vector) activities were measured and the data are represented as RLU2/RLU1. Each assay was performed in triplicate. **E.** Upregulation of miR-148/152 in Hepa cells transfected with the respective miRs compared to the controls (NC RNA transfected cells). Total RNAs from each sample in D was subjected to qRT-PCR as described in B. Each assay was performed in triplicate. **F.** Endogenous DNMT1 and DNMT3b protein levels were reduced in cells expressing ectopic miR-148/152. H293T cells were transfected with 50 nM miRs or NC RNA. After 24 h, cells were split and harvested at indicated time points post-transfection for western blot analysis of whole cell extracts (100 µg) with specific antibodies. Relative expression was determined after normalization of the signal to that of Gapdh. Single, double and triple asterisks represent P-values ≤0.05, ≤0.01 and ≤0.005, respectively.

The role of DNA methylation machinery in ALD has received little investigative attention. As a first step in understanding the role of DNA methylation machinery in alcohol-induced liver injury, we fed mice Lieber-DeCarlie liquid alcohol diet and monitored DNA methyltransferase activity, gene expression profile, global methylcytosine content and promoter methylation of selected dysregulated genes harboring CpG islands. This dietary regimen has been widely used to study alcohol-induced changes characteristic of ALD [20]. We also examined the sensitivity of Dnmt1 hypomorphic mice that express reduced level of Dnmt1 to alcohol-induced liver toxicity. This study led to some unique and rather suprising findings. First, although hepatic Dnmtase activity was significantly reduced upon feeding alcohol diet global 5-mdC level of the liver genome was not affected. Second, Dnmt1 hypomorphic (Dnmt1N/+) mice were relatively resistant to alcoholic steatosis due to altered expression of genes that are known to be involved in causing lipid accumulation and oxidative stress in the wild type mice. These distinctive observations support the notion that Dnmt1 functions as a transcriptional regulator in the terminally differentiated hepatocytes.

**Figure 3:**
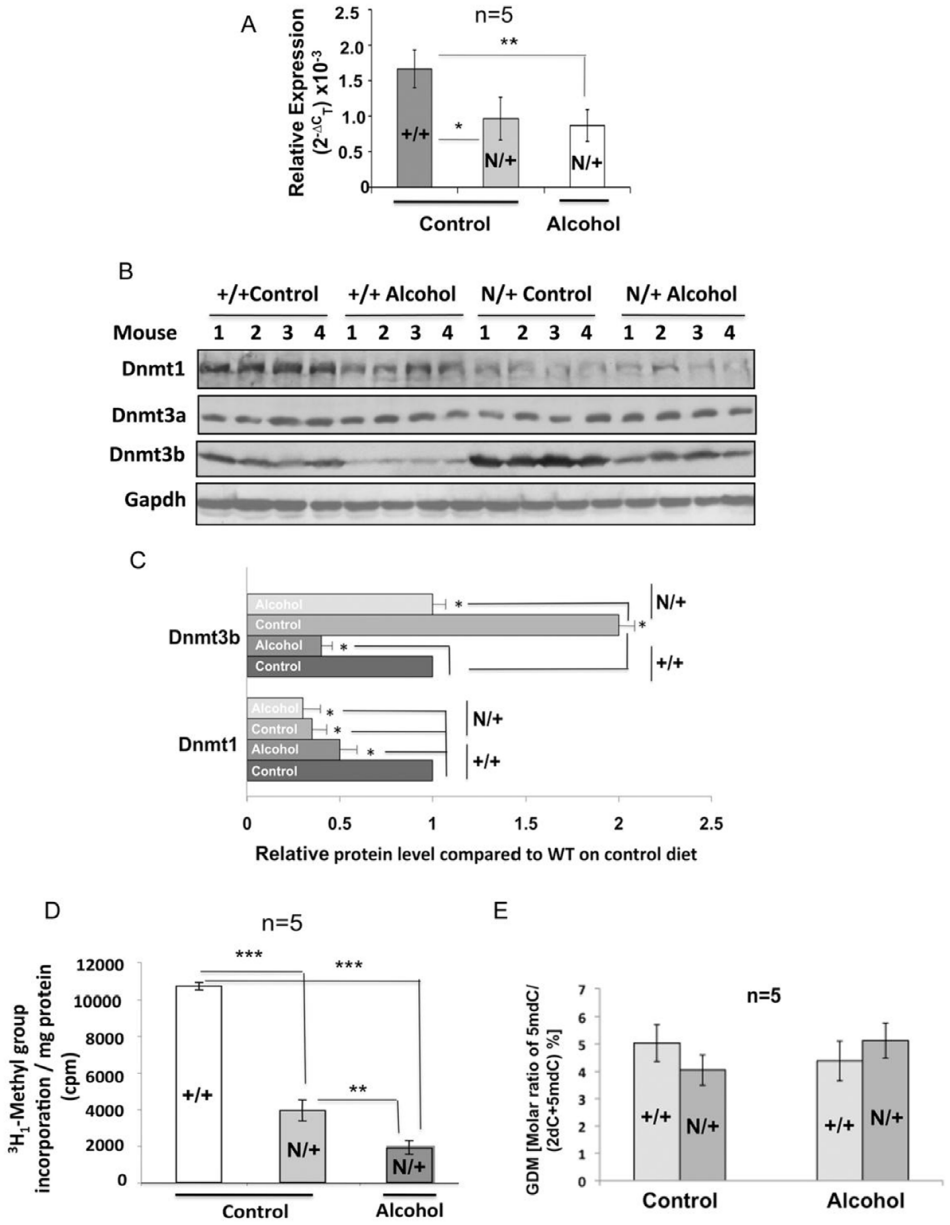
*Dnmt1* ^N/+^ mice exhibit reduced hepatic Dnmtase activity compared to the wild type mice fed control diet, which is further reduced upon feeding alcohol diet for 6 weeks. **A.** qRT-PCR analysis of Dnmt1 in the livers of the wild type (+/+) and mutant (N/+) mice. **B.** Western blot analysis of Dnmt protein levels in the liver extracts of wild type and hypomorphic mice fed control or alcohol diet. C. Quantitative analysis of the western blot data in B by ImageJ software. Gapdh normalized signal of Dnmt1 and Dnmt3b in the wild type livers was assigned a value of 1. **D.** Dnmtase activity in the hypomorphic liver nuclear extracts was significantly reduced in alcohol fed mice. Dnmtase activity was measured as described in Fig. 1A. Single, double and triple asterisks represent P-values ≤0.05≤0.01 and ≤0.005, respectively. **E** Global DNA methylation (GDM) in the liver DNA was not altered in wild type and mutant mice fed alcohol diet. Briefly, genomic DNA from the liver was enzymatically hydrolyzed to nucleosides followed by LC-MS/MS analysis. GDM is represented as a ratio of 5-mdC to total cytosine. 5mdC and 2dC denote 5-methyl-2-deoxycytidine and 2-deoxycytidine, respectively.

## Materials and Methods

### Cell Lines

Hepa1-6 (mouse hepatoma) and H293T (SV40T antigen transformed human embryonic kidney) cells were obtained from ATCC.

**Figure 4:**
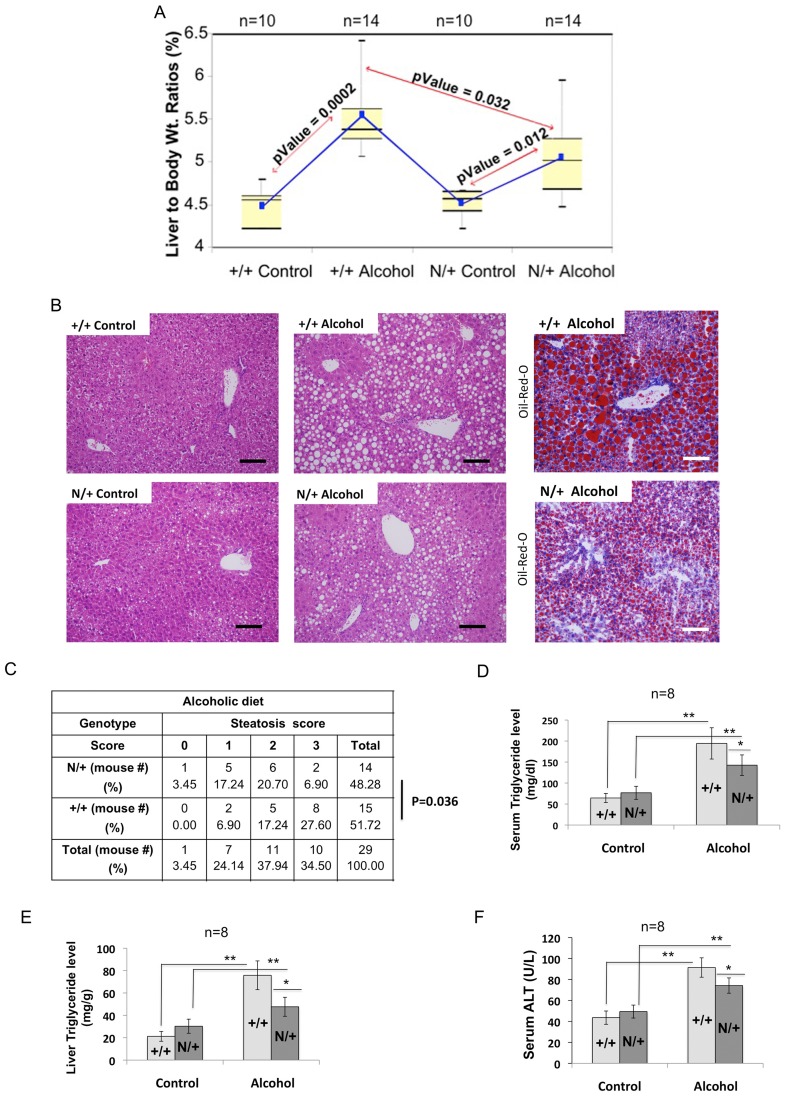
Hypomorphic mice are less sensitive than wild type mice to alcohol-induced hepatic steatosis. **A.** Alcohol-induced increase in liver weight to body weight ratio (LW/BW) is significantly less in *Dnmt1* ^N/+^ mice compared to wild type mice. Whisker-box plot of LW/BW in *Dnmt1* ^+/+^ (+/+) and *Dnmt*1^N/+^ (N/+) mice fed alcohol and pair-fed control diets for 6 weeks. The boxes show medians and upper/lower quartiles of the data, while the whiskers indicate the minimum and maximum values. The blue lines connect the mean values in consecutive boxes. **B.** Hepatic steatosis is less pronounced in *Dnmt1* ^N/+^ than in *Dnmt1* ^+/+^ mice fed the liquid alcohol diet for 6 weeks. Representative H&E and Oil-red-O stained liver sections are shown. The scale bars in the photographs are 20 µm. **C, D.** Steatosis score in mice fed the liquid alcohol (C) and pair-fed control (D) diets was determined by two blinded pathologists. Score criteria: score = 0: no steatosis; score = 1: microsteatosis; score = 2: microsteatosis and mild macrosteatosis; score = 3: severe macrosteatosis. Statistical analysis was performed using Cochran Armitage trend. **D&E.** Serum and liver triglyceride levels are significantly lower in mutant mice compared to WT mice fed the alcohol diet. Serum was collected from mice by cardiac puncture. Serum and liver TG were analyzed using the VetAce system. **F.** Alcohol-induced liver damage in *Dnmt1* ^N/+^ mice is considerably less than that in *Dnmt1* ^+/+^ mice. Serum ALT was measured using VetAce system. Statistical significance was determined by Student’s 2-tailed t test. Single and double asterisks represent P-values ≤0.05 and ≤0.01, respectively.

### Mice and Diet

All mice were housed, handled, and euthanized in accordance with NIH and institutional guidelines of the Ohio State University (OSU) Institutional Animal Care and Use Committee (IACUC). Full details of the study were approved by IACUC at OSU. The wild type (*Dnmt1* ^+/+^) and Dnmt1 hypomorphic (*Dnmt1* ^N/+^) mice [16] on a C57BL6 background were generously provided by Dr. Peter Laird at University of Southern California. Six-week old male littermates were housed in helicobacter-free facilities with 12-hour light (6 A.M. to 6 P.M.). Male mice were fed Lieber-DeCarli liquid alcohol diet purchased from BioServ following the protocol provided by the supplier. Mice were initially fed 2.06% alcohol diet for 3 days, then 4.18% alcohol diet for next 4 days followed by 6.365% alcohol diet for an additional 5 weeks. The control mice were pair-fed the control liquid diet containing maltose and dextrin for 6 weeks. 6.365% alcohol containing diet constitutes 35% of total calorie. Increases in body weight were comparable among the wild type and mutant mice on both diets.

**Figure 5:**
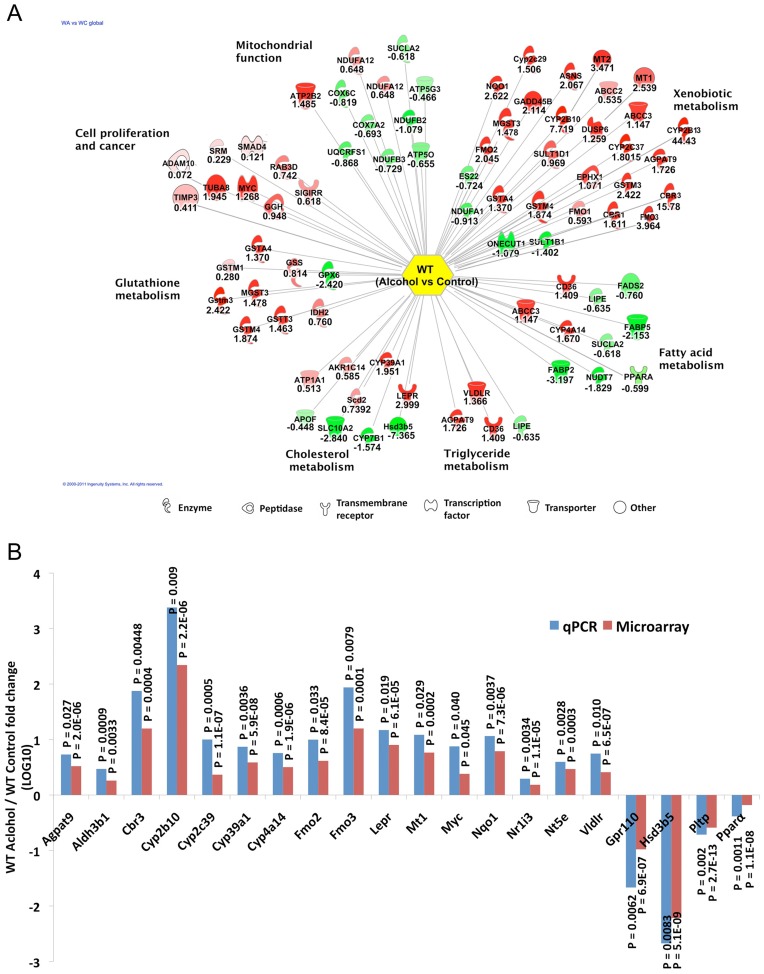
Hepatic gene expression profile is altered in the wild type mice upon feeding an alcohol-containing liquid diet for 6 weeks. **A.** IPA of the microarray data demonstrated dysregulation of genes involved in different pathways in the livers of mice fed alcohol diet. The number below each gene represents the fold change in expression (log2) in livers of mice fed alcoholic diet compared to that in controls. The shapes represent the functional class of each gene. **B.** qRT-PCR analysis validated microarray data of selected genes dysregulated in the wild type mice fed alcoholic diet compared to the controls. Fold changes in log10 scale are presented. qRT-PCR was performed with gene-specific primers using SYBR Green chemistry. Each sample was analyzed in quadruplicate.

### Serological and Histological Analysis

Mice were killed by CO_2_ asphyxiation and blood was collected by cardiac puncture. Quantification of triglyceride, cholesterol and ALT in the sera was performed using VetAce (Alfa Wassermann system) at Comparative Pathology & Mouse Phenotyping Shared Resource (OSUCCC). For histology, liver tissues were fixed in 4% para-formaldehyde (pH = 7.4) and 4 µm sections were used for H&E staining. Oil-red-O staining was performed using O.C.T. frozen tissues in the Pathology Core lab at OSU. Steatosis scoring of H&E sections was determined by two blinded pathologists following published criteria [21]; score = 0: no steatosis; score = 1: microsteatosis; score = 2: microsteatosis and mild macrosteatosis; score = 3: severe macrosteatosis. As the steatosis scores are categorical data, the Cochran Armitage trend test was used to test whether the changes in steatosis score is the same between mutant (*Dnmt1* ^N/+^) and wild type (*Dnmt1* ^+/+^) mice fed alcohol diet or pair-fed control diet.

**Table 3:** Relative expression level of several genes involved in oxidative stress response and lipid metabolic pathways in Dnmt1^N/+^ mice and Dnmt1^+/+^ mice fed alcoholic or control diet.

		Wild type (*Dnmt1* ^+/+^)		Hypomorphic (*Dnmt1* ^N/+^)	
Gene Symbol	CpG island	Control	Alcohol	Control	Alcohol
Agpat9 1-acylglycerol-3-phosphate O-acyltransferase 9	**+**	1	6*	1	1
Aldh3b1 aldehyde dehydrogenase 3 family, member B1	−	1	4*	1	6*
Myc (CMyc) v-myc myelocytomatosis viral oncogenehomolog (avian)	+	1	9*	1	2
Cyp2b10 cytochrome P450, family 2, subfamily b,polypeptide 10	−	1	4*	1	4*
Cyp2c39 cytochrome P450, family 2, subfamily c,polypeptide 39	−	1	11*	1	8*
Cyp4a14 cytochrome P450, family 4, subfamily a,polypeptide 14	−	1	6*	1	7*
Cyp39a1 cytochrome P450, family 39, subfamily A,polypeptide 1	**+**	1	7*	1	3*≠
Fmo3 flavin containing monooxygenase 3	**-**	1	119*	2	34*≠
Lepr leptin receptor	**+**	1	24*	3	4*≠
Mt1 metallothionein 1	**+**	1	21*	3	9*≠
Nqo1 NAD(P)H dehydrogenase, quinone 1	−	1	12*	2	10*
Vldlr very low density lipoprotein receptor	+	1	5*	1	2*≠
Hsd3b5 hydroxy-delta-5-steroid dehydrogenase,3 beta- and steroid delta-isomerase 5	−	1	0.002*	**0.6#**	0.002*
Ppara peroxisome proliferator-activated receptor alpha	**+**	1	0.4*	1	1

The fold changes were compared to the expression level in the wild type mice fed control diet, which was assigned an arbitrary value of 1.

qPCR analysis was performed using gene-specific primers, each sample was analyzed in triplicate and the data was normalized to 18S rRNA (n = 5). The primer sequences are provided in Table 1. Relative expression was calculated using the ΔΔC_T_ method (15). Statistical analysis of the qPCR data was performed using the student’s 2-tailed t test. * Represents significant changes in hepatic gene expression in mice fed alcoholic diet compared to those fed control diet, ≠ denotes significant changes in the hypomorphic mice compared to wild type mice both fed alcoholic diet and # indicates gene with significantly less expression in the mutant mice compared to the wild type mice both fed control diet.

### RNA, DNA and Protein Analysis

Liver tissues were snap-frozen in liquid nitrogen and stored at −80°C for later isolation of RNA, DNA and whole liver extracts [22].

### Hepatic Triglyceride and Cholesterol

Hepatic triglyceride and cholesterol were purified from the liver as described [23] and quantified using VetAce.

### DNA Methyltransferase Activity (Dnmtase) Assay

Liver nuclei were isolated from fresh tissues by sucrose density gradient centrifugation and nuclear extracts were prepared with high salt extraction following the published protocol [24]. Dnmtase activity was assayed by measuring ^3^H_1_-incorporation from ^3^H_1_-AdoMet in the substrate poly(dI-dC) as described [25]. Nuclear extracts (100 µg) were incubated with 500 ng of poly(dI-dC) 150 nM (0.55 µCi) of [methyl-^3^H_1_]-adenosylmethionine (Ado-Met) as described (24) in a total volume of 100 µl at 37°C for 1 h. Each reaction was performed in duplicate. The reaction was stopped by soaking reaction mixture onto a Whatman DE-81 ion exchange filter disc, washed (five times, 10 min each, with 0.5 M Na-phosphate buffer; pH 7.0), dried and counted in a Hitachi scintillation counter (24). The background radioactivity (without polydI-dC) was subtracted from the values obtained with reaction mixtures containing the substrate.

### Western Blot Analysis

Liver nuclear extracts were prepared by resuspending the nuclear pellet purified by sucrose density gradient in cell lysis buffer [50 mM Tris (pH 8.0), 10 mM EDTA (pH 8.0), 1% SDS containing protease inhibitor cocktail (Sigma)] and subjected to sonication to shear DNA as described [22]. Nuclear proteins were then subjected to immunoblotting with the following antibodies. Anti-Dnmt1 (1037–1386) (BioAcademia) and anti-Gapdh (MAB374) (Chemicon), respectively. Anti-Dnmt3a&3b antibodies were generated in our lab [26]. Whole liver extracts were prepared in cell lysis buffer as described above for nuclear extracts and subjected to western blot analysis with Cyp2E1 (Abcam, ab28146) and Gapdh (Chemicon, MAB374) antibodies. The signal was developed with ECL reagent (Pierce) after incubation with appropriate secondary antibodies. Western blot signals were quantified by ImageJ software (NIH) following online manual.

### Plasmid Construction, Luciferase Assays and Western Blot Analysis

The 3′-UTR of Dnmt1 was PCR amplified from mouse genomic DNA and cloned into the multiple cloning sites of psiCHECK2 (Promega), a vector that harbors both renilla (reporter) and firefly (internal control) luciferase coding regions. The primer sequences are provided in the supplementary data. The primers used for amplification of Dnmt1-3′-UTR from mouse genomic DNA. The following primers were used for cloning the region spanning Dnmt1-3′-UTR from mouse genomic DNA: mDNMT1-3′UTR-XhoI-F: 5′-CCGCTCGAGAAAGAGGAGGCTGCTACCAAGGAC-3′, mDNMT1-3′UTR-XhoI-F-del: 5′-CCGCTCGAGGAGCCCCATCATTTGAAGTCTTG-3′ and mDNMT1-3′UTR-NotI-R: 5′-ATAAGAATGCGGCCGCCACAGAAAAGTATCCCACCGAGG-3′.

Luciferase assays were performed as described [27]. Briefly, growing Hepa (hepatoma) cells (1×10^5^ cells/well), obtained from ATCC, were plated in 24-well plates the day before and transfected with 100 ng of psiCKECK2 harboring Dnmt1 3′-UTR, together with 50 nM miR-148a (Sigma) or miR-148b (Invitrogen), miR-152 (Invitrogen) mimics or respective negative control RNAs (NCRNAs) (Sigma/Invitrogen). Luciferase activities were measured 36–48 h post-transfection using dual-luciferase reporter assay system (Promega) and renilla luciferase activity was normalized to that of firefly luciferase. H293T cells obtained from ATCC, were transfected with 50 nM miR-148/152 mimic or NCRNA were harvested after 48 h and 72 h, harvested, and whole cell extracts prepared in cell lysis buffer were subjected to imunoblotting with Dnmt1,3a,3b and Gapdh antibodies.

### Global DNA Methylation Analysis

Highly purified, high molecular liver DNA was isolated as described [28] and 5 µg DNA was hydrolyzed to deoxynucleosides followed by quantification of each by liquid chromatography/mass spectrometry/mass spectrometry (LC-MS/MS) as previously described [29] and the ratio of 5methyl-deoxycytidine (5-mdC) to total cytidine (5-mdC + dC) was calculated for each sample based on the standard curve.

### Microarray Analysis

Total RNA from the livers of wild type mice fed alcohol or control diet was isolated using Trizol followed by purification using miRVana kit (Invitrogen) and the integrity and quality of the RNA was checked using an Agilent Bioanalyzer. One µg of total RNA was labeled using the Affymetrix Whole Transcript Sense Labeling protocol and was subjected to hybridization of the Affymetrix Mouse Exon 1.0 ST array following the protocol provided by the manufacturer. Microarray data are in compliance to MIAME guidelines and have been deposited to GEO database (GSE30829).

### Analysis of Microarray Data

Affymetrix GeneChip Mouse Exon 1.0 ST Array with 23,332 probe-sets was used for analysis of liver RNA from wild type mice fed alcohol diet or pair-fed control diet. 4 mice were used in each group. Signal intensities were quantified by Affymetrix software. Background correction and normalization were performed and gene expression level was summarized over probes using the RMA method [30]. A filtering method based on the percentage of samples with expression values below noise level was applied to filter out probe-sets with little or no expression, resulting in 10,430 probe-sets. Generalized linear models were used to detect differentially expressed genes between the control and alcohol diet groups. In order to improve the estimates of variability and statistical tests for differential expression, a variance smoothing method was employed [31]. The significance level was determined by controlling the average number of false positives [32]. A P-value of 0.0001 was used as the significance cutoff to allow the average number of false positives of 1.2. At least 1.5 fold increase or decrease was considered significant and used to further reduce the significant list after controlling for false positives.

### Ingenuity Pathway Analysis (IPA) of the Microarray Data

We used IPA application (http://www.ingenuity.com/products/IPA/Free-Trial-Software.html) software to analyze the microarray data. The program overlaid each identified gene from the microarray data onto its global molecular network built from IPA Knowledge Base, which is developed based on the published research. The focus genes were selected if the gene had ≥1.5 fold up or down regulation and a P-value ≤0.0001.

### Real-time RT-PCR (qRT-PCR) Analysis

The TaqMan miRNA Assay (Invitrogen) was used to quantify mature miRNAs according to manufacturer’s instructions. The expression was normalized to RNU6B. For gene expression assay, DNase I treated total RNA was reverse transcribed using high capacity cDNA reverse transcription kit (Invitrogen) and real-time PCR was performed using SYBR Green chemistry (Invitrogen). The expression was normalized to Gapdh or 18S rRNA. All real-time reactions, including controls with no templates, were run in a thermocycler in triplicate. Relative expression was calculated using the comparative method [22]. The primer sequences are provided in Table 1.

### COBRA (Combined Bisulfite Restriction Analysis) and Bisulfite Sequencing

COBRA and bisulfite sequencing were performed as described [33], [34]. Briefly, bisulfite-converted genomic DNA was PCR amplified with the gene-specific BS primers and the amplicons were digested with Tsp509 I and BstU I, Taq I or Aci I and visualized by agarose gel electrophoresis after staining with ethidium bromide. For bisulfite sequencing of *Vldlr* and *Lepr* CGI, PCR products were cloned into TA cloning vector and 5–10 randomly selected clones were sequenced. The primers for COBRA analysis of genes with CGI spanning the promoter and exon 1 are provided in Table 2.

### Statistical Analysis

Real-time RT-PCR, western blot and luciferase data were presented as means ± standard deviation (SD). Statistical significance was calculated with a Student’s two-tailed t test with a P-value of <0.05 considered significant. For non-parametric comparisons (e.g. steatosis score), Cochran-Armitage trend analysis was sued, and P≤0.05 was considered statistically significant.

## Results

### Hepatic DNA Methyltransferase Activity and Dnmt1 and Dnmt3b Protein Levels are Significantly Reduced in Mice Fed Liquid Alcohol Diet

To determine whether dietary alcohol affects DNA methylation machinery we compared Dnmtase activity in the liver nuclear extracts from mice fed liquid alcohol diet for 6 weeks (see Methods for details). The results showed that the enzyme activity was reduced by ∼50% compared to those pair-fed control diet (**Figure 1A**). The diminished Dnmtase activity correlated with ∼50% and ∼75% decrease in Dnmt1 and Dnmt3b protein levels, respectively (**Figure 1B, C**) without significant changes in respective mRNA levels (**Figure 1D, E**). While a small but significant increase in Dnmt3a RNA level was observed in response to alcohol diet, its protein level was not significantly altered (**Figure 1B, F**). Thus, the reduced Dnmt1 and Dnmt3b protein levels probably contributed to decrease in hepatic DNMTase activity in mice fed alcohol diet.

### miR-148 and miR-152 that Target Dnmt1 and Dnmt3b are Upregulated in the Livers of Mice Fed Alcohol Diet

Next, we sought to elucidate the mechanism underlying alcohol-induced suppression of Dnmt1 and Dnmt3b proteins. We entertained the possible involvement of microRNAs because mRNA levels of these two enzymes were not significantly altered in the livers of alcohol fed mice (**Figure 1D, E**). A TargetScan database (http://www.targetscan.org/mmu_50/) [35] search revealed that 3′-UTR of Dnmt1 harbors only one conserved site for miR-148 and miR-152 (**Figure 2A**). Notably, both of these miRs have identical seed sequence implying that these miRs target common mRNAs. Interestingly, miR-148 has been shown to target Dnmt3b by complementary base pairing with two conserved sites located in its coding region [36]. To determine if these miRs play any role in the suppression of hepatic Dnmt1 and Dnmt3b levels in alcohol fed mice, we first measured expression of these two miRs. Northern blot analysis showed that miR-148 was elevated in all 6 mice fed the liquid alcohol diet, albeit at different levels, compared to those fed control diet (**Figure 2B**). qRT-PCR analysis confirmed 40% increase in miR-148 level whereas miR-152 expression (not detectable by Northern blotting) increased by 85% in the livers from mice fed the liquid alcohol diet compared to controls (**Figure 2C**). These results suggest that co-ordinate upregulation of miR-148/152 is likely to be involved in down regulation of hepatic Dnmt1/3b in alcohol fed mice.

We then investigated whether Dnmt1 is a target of miR-148 and/or miR-152. For this purpose, we cloned 3′-UTR of Dnmt1 into psiCHECK2 vector downstream of the renilla luciferase coding region and transfected it in Hepa cells along with miR-148b, miR-152 or negative control RNA (NC RNA). After 48 h, cells were assayed for relative luciferase (renilla/firefly) activity and miR-148b/152 expression. The results showed that miR-148b and miR-152 could reduce Dnmt1 3′-UTR driven renilla luciferase (RLU2) activity by 20% and 45%, respectively (**Figure 2D**). The lack of inhibition of relative luciferase activity in cells transfected with the mutant psiCHECK2-Dnmt1-3′UTR vector lacking the miR-148/152 cognate site validated Dnmt1 as a true target of these two miRs. Relatively less pronounced inhibition of reporter activity in miR-148b transfected cells compared to those transfected with NC RNA is probably due to higher miR-148b level in these cells, which was elevated only 2 fold upon transfecting miR-148b mimic (**Figure 2D, E**). Similar results were obtained after ectopic expression of miR-148a (data not shown). More robust inhibition (45%) of Dnmt1-3′UTR driven RLU2 activity in miR-152 transfected cells correlated with low basal expression of this miR in these cells which increased ∼500 - fold upon ectopic miR-152 expression (**Figure 2D, E**).

Next, we examined whether endogenous Dnmt1 and Dnmt3b protein level could be modulated by miR-148 and miR-152 by transfecting these miRs or respective NC RNAs into H293T cells. These cells were chosen for this assay because of much higher transfection efficiency compared to Hepa cells. Western blot analysis showed that ectopic miR-148b reduced DNMT1 protein level by ∼40% and ∼50% 48 h and 72 h post-transfection, respectively, compared to the controls (**Figure 2F**). Ectopic miR-152 reduced Dnmt1 level by ∼30% at both time points whereas the DNMT3b level was reduced (∼30%) by both miRs only after 72 h. The differential effects of miR-148 and miR-152 on Dnmt1 and Dnmt3b protein compared to 3′-UTR-driven luciferase assay may be due to different cell types used. Alternatively, the function of 3′-UTR in the context of homologus mRNA may not be identical when it is linked to a heterologus mRNA (renilla luciferase). Taken together, these results suggest that upregulation of both miR-148 and miR-152 plays a causal role in suppressing their common targets, Dnmt1 and Dnmt3b, in mice fed a liquid alcohol diet.

### Dnmt1 Hypomorphic Mice Exhibit Resistance to Alcohol-induced Hepatic Steatosis Compared to the Wild Type Mice

To determine the consequence of significant decrease in Dnmtase activity on alcohol-induced liver pathogenesis, Dnmt1 hypomorphic mice (*Dnmt1* ^N/+^) that express reduced levels of Dnmt1 [16], were fed control and alcohol diets. Dnmt1 expression is reduced in these mice by ∼50% due to disruption of one allele by homologus recombination [16]. qRT-PCR and Western blot analysis showed ∼50% and ∼60% reduction in hepatic Dnmt1 RNA and protein levels, respectively in *Dnmt1* ^N/+^ mice compared to *Dnmt1* ^+/+^ mice fed control diet (**Figure 3A, B**). However, Dnmt1 level was not measurably reduced further upon feeding the alcohol diet. Surprisingly, hepatic Dnmt3b protein level was elevated by ∼50% in the mutant mice compared to the wild type mice fed control diet (**Figure 3B, C**). As observed in the wild type mice, feeding the liquid alcohol diet reduced Dnmt3b level by ∼50% in hypomorphic mice. Dnmt3a protein level was comparable in *Dnmt1* ^+/+^ and *Dnmt1* ^N/+^ mice irrespective of the diet (**Figure 1B** **and** **3B**). Analysis of hepatic Dnmtase activity in the nuclear extracts showed ∼60% and ∼80% reduction in hypomorphic mice fed control and alcohol diets, respectively, compared to the wild type mice fed control diet (**Figure 3D**). Hepatic Dnmtase activity in alcohol fed *Dnmt1* ^N/+^ mice was ∼50% less than that of *Dnmt1* ^+/+^ mice fed alcohol diet (compare **Figure 3D** and **Figure 1A**). However, analysis of global DNA methylation (GDM) by LC-MS/MS showed that hepatic 5-methyldeoxycytidine content in *Dnmt1* ^+/+^ and *Dnmt1* ^N/+^ mice fed control diet was comparable and was not significantly affected by dietary alcohol in either genotype (**Figure 3E**). These results suggest that 50% of the Dnmtase activity is sufficient to maintain GDM in the livers of *Dnmt1* ^N/+^ mice and feeding alcohol did not purturb it in the adult liver.

We next compared the phenotype of *Dnmt1* ^+/+^ and *Dnmt1* ^N/+^ mice fed alcohol and control diets for 6 weeks. Increase in body weight and food consumption was comparable between the two genotypes and two diet groups (data not shown). As expected, the liver to body weight ratios (LW/BW) increased significantly in both genotypes fed the alcohol diet compared to those pair-fed the control diet (**Figure 4A**). The alcohol-induced increase in LW/BW in the mutant mice was, however, significantly less than that in the wild type mice.

Analysis of H&E stained liver sections showed that mice fed alcohol diet developed steatosis characterized by numerous vacuoles in the liver (representative photographs are shown in **Figure 4B**). Oil-Red-O staining confirmed that these vacuoles were lipid droplets. Both H&E and Oil-red-O staining of these representative slides revealed that steatosis in hypomorphic mice was less pronounced than that in the wild type mice fed alcohol. Blinded scoring of steatosis in 15 mice from two different experiments showed a significant difference in the steatosis score distribution between the wild type and mutant mice fed alcohol diet (P = 0.036) (**Figure 4C**), indicating that the steatosis-score was highly correlated with the genotype. However, analysis of mice pair-fed control diet did not reveal significant association between steatosis-score and genotype (P = 0.58) (**Figure 4D**). Both *Dnmt1* ^+/+^ and *Dnmt1* ^N/+^ mice fed alcohol developed mild hepatic inflammation as demonstrated by infiltration of inflammatory cells (**Figure 4B**).

We next assessed serum and hepatic triglyceride (TG) level since alcohol is known to cause elevation in TG content that leads to steatosis [1]. Both serum and hepatic TG levels increased in the wild type and mutant mice fed alcohol (**Figure 4E, F**). However, the increase in serum and liver TG levels in hypomorphic mice was ∼30% and ∼50%, respectively of that in the wild type mice. Reduced hepatic TG accumulation in hypomorphic mice fed the liquid alcohol diet correlated with comparatively diminished steatosis in these mice (**Figure 4B, C**). In contrast, alcohol-induced increase in cholesterol level was comparable among the mice of two genotypes (data not shown). To assess liver damage in these mice we measured serum ALT level which showed ∼2-fold increase in the wild type mice fed alcohol compared to those fed control diet, whereas it increased only 1.6 fold in *Dnmt1* ^N/+^ mice fed alcohol diet (**Figure 4G**). Taken together, these results demonstrate that in spite of decrease in hepatic Dnmtase activity in mice fed the alcohol diet, *Dnmt1* hypomorphic mice are relatively resistant to alcohol-induced steatosis and liver damage compared to the wild type mice.

### A Large Number of Genes Involved in Metabolic Pathways are Differentially Expressed in the Wild Type Mice Fed Alcohol Diet

To elucidate the mechanism by which alcohol affects liver physiology we compared hepatic gene expression profiles in the wild type mice fed control and alcohol diets by microarray analysis (see Methods for details). The results showed that 98 genes were upregulated (≥1.5 fold, P≤0.0001) and 102 genes were downregulated (≤0.67 fold, P≤0.0001) upon feeding alcohol (**Figure S1**). Principal component analysis of the microarray data classified mice into two groups based on the diet (**Figure S2**). Ingenuity Pathway Analysis (IPA) [www.ingenuity.com] showed that the majority of the dysregulated genes encode enzymes that are involved in several metabolic pathways including xenobiotic, glutathione and lipid (triglyceride, fatty acid and cholesterol) metabolism (**Figure 5A**). Increased oxidative stress generated by acetaldehyde, a highly reactive alcohol metabolite, is likely to cause dysregulation of mitochondrial and microsomal genes [37]. Indeed, chronic alcohol feeding significantly elevated hepatic *Cyp2e1* (oxidizes ethanol to acetaldehyde) at the protein level without altering its RNA level (**Figure S3**). Intriguingly, alcohol feeding also induced expression of several genes including *C-Myc, Timp3, Adam-10* that are known to be involved in cell proliferation/tumorigenesis (**Figure 5A**), although feeding alcohol for 6 weeks did not cause proliferation of hepatoctyes (data not shown). It is of interest to note that C-Myc is also a master regulator of metabolism [38].

Several genes upregulated following alcohol feeding are involved in the oxidative stress response, which include *Cyp2b10* (220 fold), *Cyp2b13* (44 fold), *Fmo3* (16 fold), *Cbr3* (16 fold), *Nqo1* (6 fold) (**Figure 5A**). Similarly, several genes encoding glutathione metabolic enzymes except *Gpx6* were upregulated in mice fed alcohol. In contrast, genes involved in mitochondrial function, fatty acid and cholesterol metabolism were both positively and negatively regulated in response to alcohol feeding. Expression of several factors that are known to regulate lipid metabolism and transport, such as *Agpat9* (1-acylglycerol-3-phosphate O-acyltransferase 9) (3.3 fold), *Vldlr* (very low density lipoprotein receptor) (2.6 fold) and *Scd2* (stearoyl-Coenzyme A desaturase 2) (1.6 fold) increased whereas that of *Ppara* (peroxisome proliferator-activated receptor alpha), a key transcription factor with a protective role against ALD [39] decreased by 64% in the livers from alcohol fed wild type mice.

Next, we validated microarray data on selected genes. The results showed that 16 genes upregulated in the microarray analysis were even more elevated by qRT-PCR analysis (**Figure 5B**). Similarly, downregulation of 4 selected genes in alcohol fed mice was more pronounced after validation by PCR analysis. Taken together, these results revealed modulation of expression of hepatic genes involved in various metabolic pathways in response to dietary alcohol and supported the contention that dysregulation of some of these genes may play a causal role in steatosis.

### Differential Expression of Genes Involved in Metabolic Pathways in Hypomorphic Mice Fed Alcohol Diet

To delineate the mechanism for reduced steatosis and liver damage in *Dnmt1* ^N/+^ mice fed the alcohol diet, we next compared the expression of several genes identified by microarray in the wild type and mutant mice. qRT-PCR analysis showed that 7 genes (*Aldh3b1, Cyp39a1, Fmo3, Lepr, Mt1, Vldlr* and *Scd2*) were upregulated in both genotypes fed alcohol, albeit at different levels, whereas the inductions of *Cyp2b10, Cyp2c39, Cyp4a14, Nqo1* and *Aldh3b1* were comparable between the two genotypes (**Table 3**). However, induction of *Cyp39a1* (7 vs 3 fold), *Fmo3* (119 vs 34 fold), *Vldlr* (5 vs 2 fold) and *Mt1* (21 vs 9 fold) were less pronounced in the mutant livers. Notably, *Agpat9* (6 fold), *Lepr* (24.5 fold) and *c-Myc* (9 vs 2 fold) were significantly induced only in the wild type mice fed alcohol. The level of *Ppara* mRNA was reduced by 60% only in the wild type mice fed alcohol and remained unaltered in hypomorphic mice. In contrast, the expression of hepatic *Hsd3b5* was relatively high in mice fed the control diet, which was dramatically suppressed (>400 fold) in alcohol fed mice of both genotypes.

We then addressed the potential involvement of DNA methylation in alcohol induced dysregulation of some of these genes in the livers of *Dnmt1* ^+/+^ and *Dnmt1* ^N/+^ mice. Among these genes CpG islands (CGIs) spanning the promoter and/or exon1 of *Agpat9*, *c-Myc*, *Lepr, Vldlr, Cyp39a1, Mt1* and *Ppara* were identified by BLAT search (http://genome.ucsc.edu/cgi-bin/hgGateway?org=Mouse&db=mm9&hgsid=224384625). However, combined bisulfite restriction analysis (COBRA) and bisulfite sequencing did not reveal any detectable methylation at these CGIs in any of the four groups (2 diets and 2 genotypes) of mice (data not shown) suggesting that promoter methylation is not involved in regulating expression of these genes in mouse livers (data not shown). These results imply that alcohol induced expression of these genes is likely to be regulated by transcriptional activation and/or chromatin modification.

## Discussion

It is now well established that like transcription factors, epigenetic modifications of the DNA and histones play a major role in gene regulation and that aberrations in the epigenetic machinery can cause multiple diseases such as cancer, neuronal, immunological, and metabolic disorders [40]. The purpose of this study was to determine whether chronic alcohol feeding modulates the expression/activity of DNA methylation machinery and whether reduced expression of Dnmt1 has any effect on alcohol-induced liver dysfunction. Our study revealed that hepatic Dnmtase activity was reduced upon chronic alcohol feeding, which correlated with reduced expression of Dnmt1 and Dnmt3b protein but not RNA levels. We also showed that alcohol-induced upregulation of both miR-148 and miR-152 play a key role in downregulating their common targets Dnmt1 and Dnmt3b. We focused on miR-148/152 because only these two miRs have a common site on Dnmt1 3′-UTR, which is conserved in mammals, and miR-148 and miR-152 have been previously shown to target Dnmt1 by interacting through its 3′-UTR [41], [42], whereas miR-148 targets Dnmt3b by binding to its cognate site in the coding region [36]. Decrease in Dnmt1 and Dnmt3b expression and Dnmtase activity suggested to us that Dnmt1 hypomorphic mice might be more susceptible to alcoholic liver disese. Surprisingly, compared to the wild type mice these mice were relatively resistant to hepatic steatosis, as shown by reduced accumulation of triglyceride and expression of several key enzymes and transcription factors involved in lipid metabolism, oxidative stress that are associated with ALD These observations imply that *Dnmt1^N^* allele exhibits protective role not only in colon tumorigenesis mice [15], [16] but also in alcohol-induced hepatosteatosis.

In cancer cells loss of Dnmtase activity and suppression of Dnmt1 and Dnmt3b lead to passive loss of global hypomethylation during rapid proliferation [43]. However, analysis of global DNA methylation (GDM) by LC-MS/MS showed that relative 5-methyldeoxycytosine content in the liver was not significantly affected by alcohol in the *Dnmt1* ^+/+^ or *Dnmt1* ^N/+^ mice (**Figure 3E**). This is not surprising considering the fact that the hepatocytes in the adult mice are in terminally differentiated state and feeding alcohol diet for 6 weeks did not cause these cells to enter cell cycle (data not shown). Unabated expression of Dnmt3a may be adequate for maintaining the methylation profile in nonreplicating hepatocytes. Although unlikely, we cannot rule out active demethylation of some genes in the livers of mice after feeding alcohol. Genome wide approaches using recently developed techniques [44] is likely to identify such genes. It is also possible that chronic alcohol intake alone or in combination with carcinogen exposure may result in differential methylation of metabolic and tumor causing genes. Prolonged alcohol exposure could also result in altered expression of genes that are distinct from short term feeding of alcohol diet as used in the present study. It would be of interest to investigate the liver methylome in mice and humans with chronic alcoholic liver disease.

The resistance of *Dnmt1* ^N/+^ mice to alcohol-induced steatosis merits discussion. This phenotype could be explained by differential expression of some key genes involved in lipid metabolic pathways. First, *Agpat9*, an enzyme in triglyceride biosynthesis, is upregulated only in the wild type mice fed alcohol (**Table 3**). Second, *Ppara*, a nuclear hormone receptor that modulates transcription of several genes involved in fatty acid transport and oxidation [45], is dramatically downregulated only in the wild type mice fed alcohol (**Table 3**). It has been shown that Ppara *null* mice are sensitive to alcoholic liver disease [46]. It is conceivable that persistent expression of *Ppara* in *Dnmt1* ^N/+^ livers could contribute to reduced steatosis in these mice fed alcohol diet. Third, elevated expression (>2-fold) of *Aldh3b1* that detoxifies acetaldehyde to acetate in hypomorphic mice compared to the wild type mice suggests reduced oxidative stress in the hypomorphic mice. Absence of detectable methylation in the CGI of these genes in both genotypes fed alcohol or control diet indicates that DNA methylation may not directly regulate expression of these genes. However, we cannot rule out the possibility that Dnmt1 predominantly functions as a transcriptional regulator in the terminally differentiated hepatocytes. Indeed, all three Dnmts can function as transcriptional repressors that require their N-terminal regulatory domains but not its C-terminal catalytic domains [13], [14]. It is therefore, possible that the reduced Dnmt1 level in concert with alcohol and/or its metabolites can cause derepression of one or more factors in the hypomorphic mice, which may be responsible for differential expression of genes such as *Aldh3b1, Agpat9, c-Myc, Fmo3, Lepr* and *Vldlr*. Additionally, altered chromatin structure of some of these genes due to changes in the histone code of associated nucleosomes in the hypomorphic mice may also contribute to the resistance of these mice to alcohol-induced liver pathogenesis. Relatively low level induction of *Fmo3* and *Mt1* suggests that overall oxidative stress due to alcohol feeding is less in *Dnmt1* ^N/+^ mice compared to the wild type mice, thereby accounting for reduced pathogenesis. Finally, alcohol may differentially affect liver metabolites in the hypomorphic mice compared to the wild type mice. Future studies on the liver transcriptome, epigenome and metabolome will be able to elucidate the detailed mechanisms by which Dnmt1 modulates ALD.

## Supporting Information

Figure S1 **Volcano plot demonstrating genes up and down regulated (** ***P*** **≤0.0001) in the wild type mice upon feeding alcoholic compared to the control diet.** Liver RNA from 4 mice in each diet group was subjected to mouse exon array (see Methods for details).
(DOCX)

Figure S2 **Principal Component Analysis discriminated mice fed alcohol and control diet groups based on gene expression profiles.** The expression data of all genes on the microarray were projected in three dimensions (PC1, PC2 and PC3) while keeping 56% variation in the data. PC1 - PC3 are the combination of expression of all genes.
(DOCX)

Figure S3 **Hepatic Cyp2e1 protein and mRNA levels were measured by immunoblot and real-time RT-PCR analysis in the wild type (+/+ or WT) and Dnmt1 hypomorphic (N/+) mice fed alcoholic or and control diet for 6 weeks.** The data was normalized to Gapdh level. Each assay was performed in triplicate (n = 5).
(DOCX)
