# Supplementary figures and images for: Reduced Susceptibility of DNA Methyltransferase 1 Hypomorphic (Dnmt1N/+) Mice to Hepatic Steatosis upon Feeding Liquid Alcohol Diet
Source: PLoS One. 2012 Aug 8;7(8):e41949. doi: 10.1371/journal.pone.0041949 (PMC3414497; doi:10.1371/journal.pone.0041949)

**Figure S1**


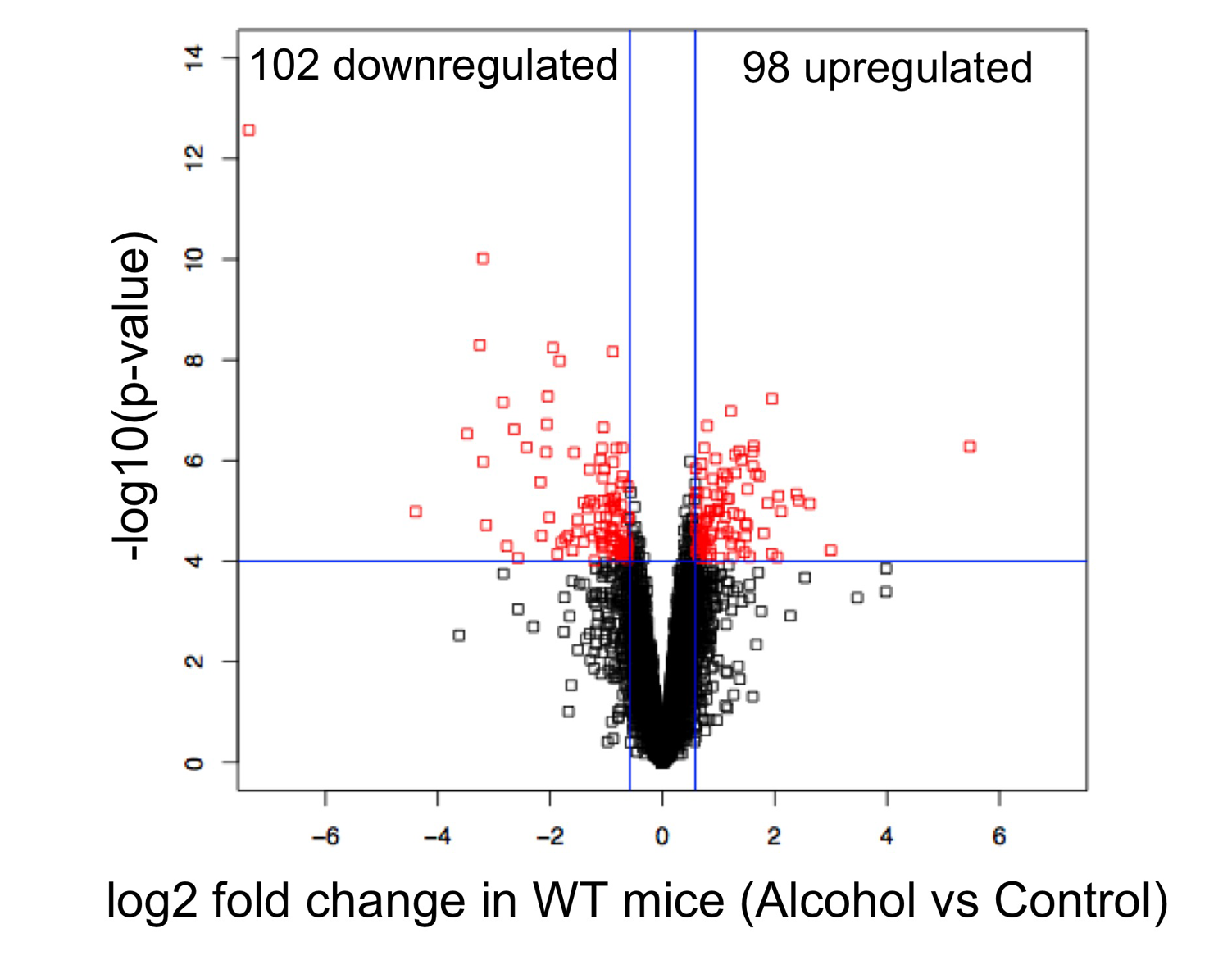

Supplement: Figure S1 — Volcano plot demonstrating genes up and down regulated ( P ≤0.0001) in the wild type mice upon feeding alcoholic compared to the control diet. Liver RNA from 4 mice in each diet group was subjected to mouse exon array (see Methods for details). (DOCX) [file pone.0041949.s001.docx]

**Figure S2**


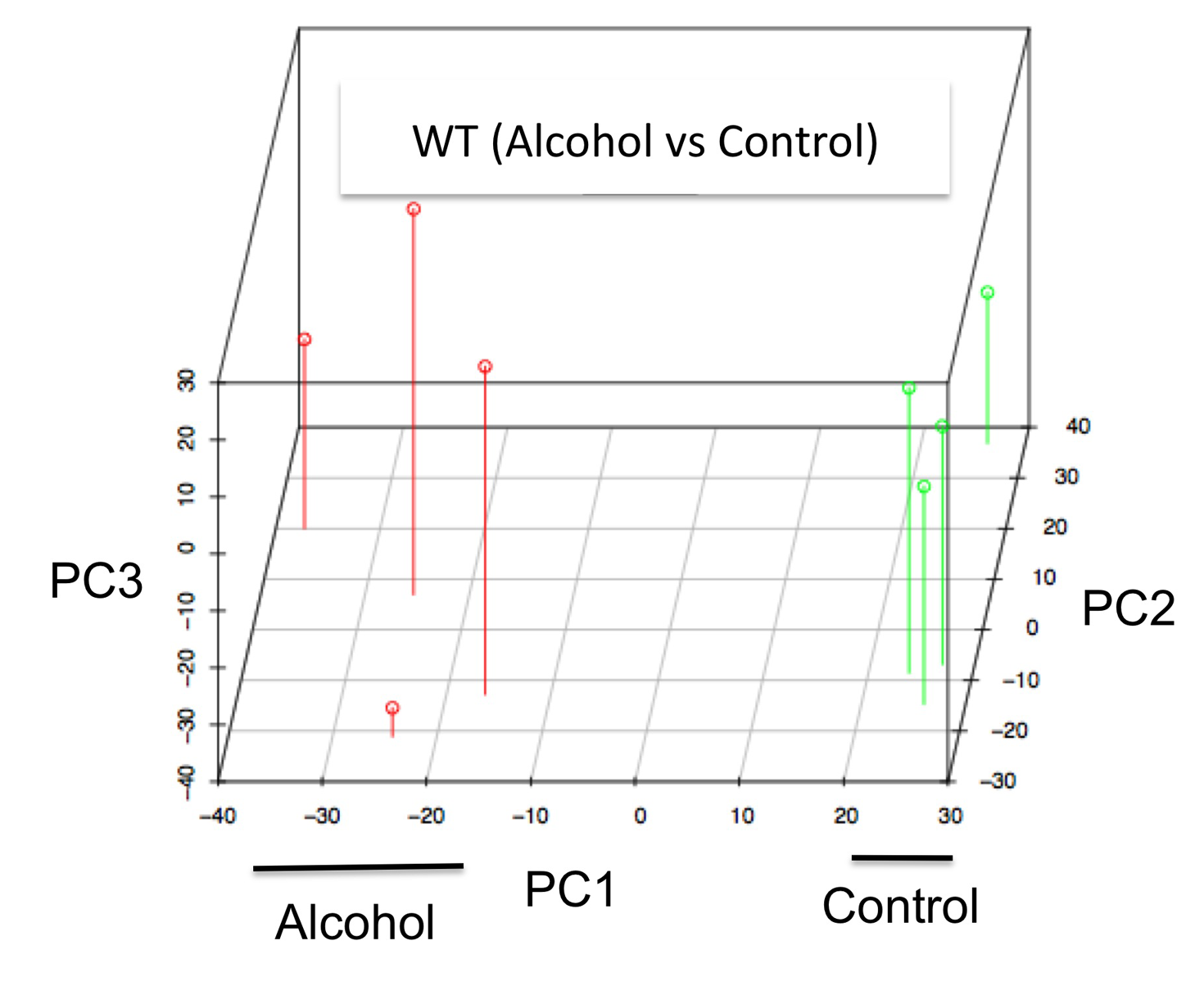

Supplement: Figure S2 — Principal Component Analysis discriminated mice fed alcohol and control diet groups based on gene expression profiles. The expression data of all genes on the microarray were projected in three dimensions (PC1, PC2 and PC3) while keeping 56% variation in the data. PC1 - PC3 are the combination of expression of all genes. (DOCX) [file pone.0041949.s002.docx]

**Figure S3**


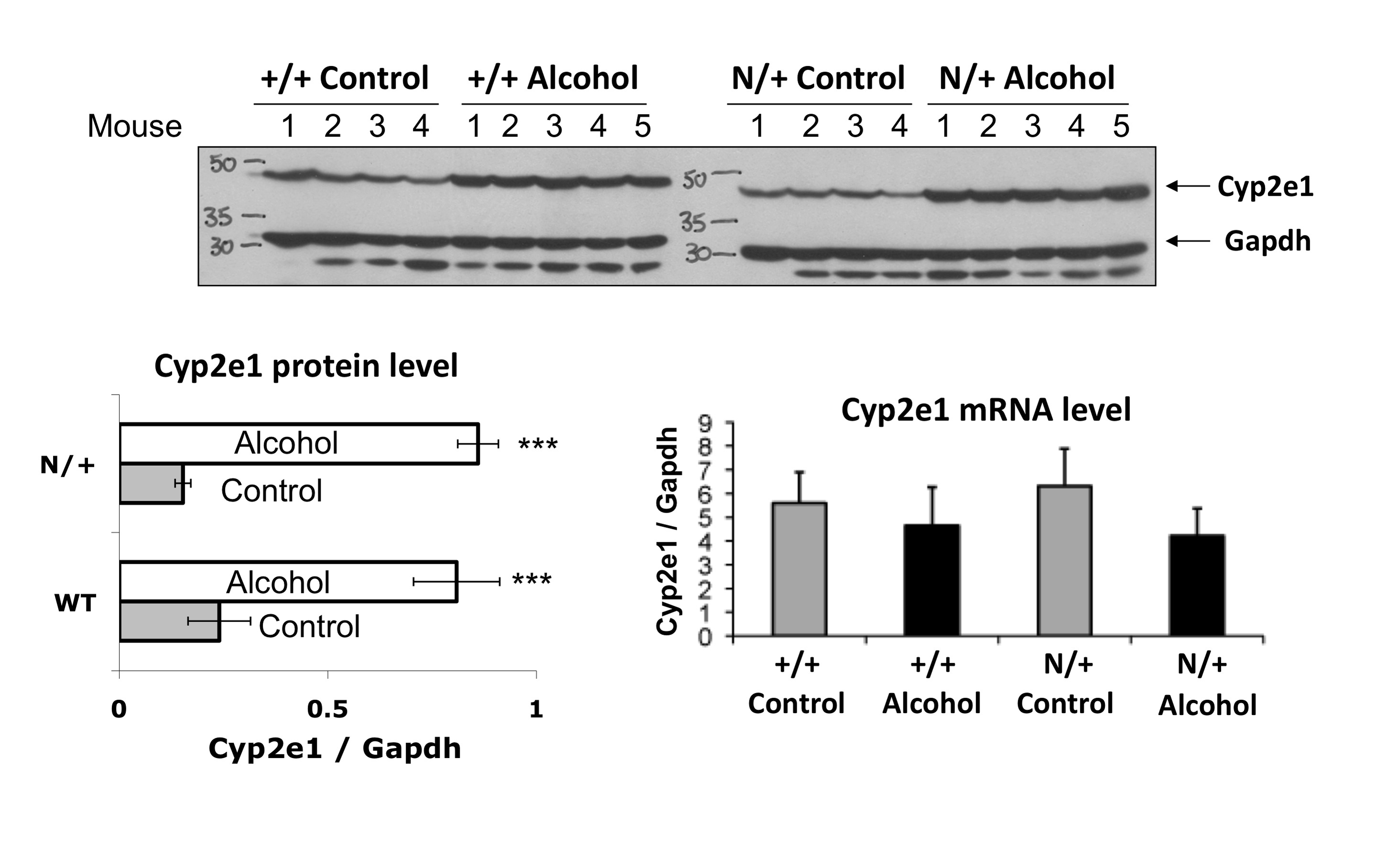

Supplement: Figure S3 — Hepatic Cyp2e1 protein and mRNA levels were measured by immunoblot and real-time RT-PCR analysis in the wild type (+/+ or WT) and Dnmt1 hypomorphic (N/+) mice fed alcoholic or and control diet for 6 weeks. The data was normalized to Gapdh level. Each assay was performed in triplicate (n = 5). (DOCX) [file pone.0041949.s003.docx]
